# Supplementary material for: Identifying the Vertical Stratification of Sediment Samples by Visible and Near-Infrared Spectroscopy
Source: Sensors (Basel). 2024 Oct 14;24(20):6610. doi: 10.3390/s24206610 (PMC11511207; doi:10.3390/s24206610)
Supplement: Supplementary file 1 [file sensors-24-06610-s001.zip › sensors-3208238-supplementary.pdf]

**Table S1.** The specifications of spectrometers Cary 5000.

| Features | Cary 5000 |
|---|---|
| Sensor | photodiode and TE cooled PbS |
| Detector | quartz window |
| Wavelength range | 350-2500 nm |
| Optical resolution | 1 nm |
| Signal to noise ratio | >30000 |
| Integration time | 100 ms |
| Stray light | <0.0002% (1420nm) |
| Wavelength repeatability | <0.02nm (>750nm); <0.005nm (<750nm) |
| Wavelength accuracy | <0.4nm (>750nm); <0.08nm (<750nm) |
